# Open lung biopsy in early-stage acute respiratory distress syndrome

**DOI:** 10.1186/cc4981

**Published:** 2006-07-19

**Authors:** Kuo-Chin Kao, Ying-Huang Tsai, Yao-Kuang Wu, Ning-Hung Chen, Meng-Jer Hsieh, Shiu-Feng Huang, Chung-Chi Huang

**Affiliations:** 1 Division of Pulmonary and Critical Care Medicine, Chang Gung Memorial Hospital, Chang Gung University College of Medicine, 5 Fu-Hsin Street, Kweishan, Taoyuan 333, Taiwan; 2 Department of Respiratory Therapy, Chang Gung Memorial Hospital, 5 Fu-Hsin Street, Kweishan, Taoyuan 333, Taiwan; 3 Department of Pathology, Chang Gung Memorial Hospital, Chang Gung University College of Medicine, 5 Fu-Hsin Street, Kweishan, Taoyuan 333, Taiwan; 4 Division of Molecular and Genomic Medicine, National Health Research Institute, 35 Keyan Road, Zhunan, Miaoli 350, Taiwan

## Abstract

**Introduction:**

Acute respiratory distress syndrome (ARDS) has heterogeneous etiologies, rapid progressive change and a high mortality rate. To improve the outcome of ARDS, accurate diagnosis is essential to the application of effective early treatment. The present study investigated the clinical effects and safety of open lung biopsy (OLB) in patients with early-stage ARDS of suspected non-infectious origin.

**Methods:**

We undertook a retrospective study of 41 patients with early-stage ARDS (defined as one week or less after intubation) who underwent OLB in two medical intensive care units of a tertiary care hospital from 1999 to 2005. Data analyzed included baseline characteristics, complication rate, pathological diagnoses, treatment alterations, and hospital survival.

**Results:**

The age of patients was 55 ± 17 years (mean ± SD). The average ratio of arterial partial pressure of oxygen (PaO_2_) to fraction of inspired oxygen (FiO_2_) was 116 ± 43 mmHg (mean ± SD) at biopsy. Seventeen patients (41%) were immunocompromised. Postoperative complications occurred in 20% of patients (8/41). All biopsies provided a pathological diagnosis with a diagnostic yield of 100%. Specific pathological diagnoses were made for 44% of patients (18/41). Biopsy findings led to an alteration of treatment modality in 73% of patients (30/41). The treatment alteration rate was higher in patients with nonspecific diagnoses than in patients with specific diagnoses (*p *= 0.0024). Overall mortality was 50% (21/41) and was not influenced by age, gender, pre-OLB oxygenation, complication rate, pathological results, and alteration of treatment. There was no surgery-related mortality. The survival rate for immunocompromised patients was better than that for immunocompetent patients (71% versus 33%; *p *= 0.0187) in this study.

**Conclusion:**

Our retrospective study suggests that OLB was a useful and acceptably safe diagnostic procedure in some selected patients with early-stage ARDS.

## Introduction

The clinical definition of acute respiratory distress syndrome (ARDS) includes the acute onset of bilateral pulmonary infiltrates, a ratio of arterial partial pressure of oxygen (PaO_2_) to fraction of inspired oxygen (FiO_2_) of 200 mmHg or less, and no evidence of left atrial hypertension [1]. Many risk factors, such as pneumonia, sepsis, and aspiration, are associated with the development of ARDS. However, other diseases and conditions, such as bronchiolitis obliterans organizing pneumonia (BOOP), adverse reaction to drugs, diffuse alveolar hemorrhage (DAH), and hypersensitivity pneumonitis (HP), can also cause ARDS; despite similar clinical presentations, etiological diagnosis can be difficult especially for early-stage ARDS. Although the mortality rate of patients with ARDS improves recently [2], the rapid clinical deterioration of such patients, who often progress to multiple organ failure, remains a significant challenge for intensivists in the intensive care unit (ICU). To halt the disease progression of early-stage ARDS, accurate diagnosis is critical.

It can be difficult to differentiate between infectious and non-infectious etiology as the cause of ARDS in its early stages. Current microbiological sampling techniques are insufficiently sensitive to determine the causes of ARDS in all patients [3-5]. In patients with negative microbiological cultures, separating a true infection from an inflammatory response with clinical data remains problematic. Empiric broad-spectrum antibiotics are typically prescribed to these critically ill patients immediately after admission. However, unnecessary antibiotic therapy for non-infectious patients can enhance the occurrence of antibiotic-resistant strains of bacteria and increase the potential for subsequent nosocomial infections.

The therapeutic benefit of prolonged glucocorticoid therapy during the fibroproliferative stage of ARDS emphasizes the need for the elucidation of the underlying lung pathologies [6]. Additionally, the specific diseases such as BOOP, drug reaction, DAH and HP can cause an ARDS response to steroid therapy. However, inappropriate steroid therapy for patients with ARDS may be associated with complications such as gastrointestinal bleeding, hyperglycemia and increased susceptibility to infection.

Some previous studies have demonstrated that open lung biopsy (OLB) is a useful and acceptably safe diagnostic technique for patients with ARDS [7-9]. In the study by Papazian and colleagues [7], the results of OLB directly altered the therapeutic management for 34 of 36 patients with ARDS (94%), and the OLB complication of an air leak occurred in five patients (14%). The OLB results obtained by Patel and colleagues [8] led to a change in management in the majority of 57 patients with ARDS, the addition of specific therapy for 34 patients (60%), and the withdrawal of unnecessary therapy in 24 patients (37%); major complications occurred in four patients (7%). However, in both studies the duration from intubation to OLB was long: in Papazian and colleagues' study [7] the range was 5 to 89 days, and in Patel and colleagues' study [8] it was 0 to 25 days.

This retrospective study attempted to evaluate the utility and safety of OLB in patients with clinically suspected non-infectious early-stage ARDS.

## Methods

### Patients

The records of patients with ARDS who received OLB in two ICUs at a tertiary care referral center over a five year period between January 1999 and April 2005 were examined. Charts with a discharge diagnosis code 518.82 of the International Classification of Diseases, Ninth Revision, Clinical Modification, suggesting ARDS not related to surgery or trauma, were reviewed for possible inclusion in this study. A total of 819 patients with ARDS were identified and OLBs were performed in 68 patients (8.3%). Forty-one OLBs were performed during early-stage ARDS (one week or less after intubation). Patients supported with noninvasive positive-pressure ventilation or intubated for more than seven days at the time of biopsy were excluded.

All patients met ARDS criteria defined by the American-European consensus conference [1]. Decisions to perform OLB were made by senior intensivists in charge of the respective ICUs. OLB was indicated when ARDS was suspected to be noninfectious in origin, with no obvious etiology and with a possible indication for corticosteroid treatment based on clinical presentations with rapid progression, relative symmetric distribution on chest X-ray, and predominant ground-glass attenuation in high-resolution computed tomography (HRCT) of the chest. Informed consent for OLB was obtained from each patient's family.

### Radiological and microbiological examinations performed before open lung biopsy

Chest HRCT was performed before bronchoscopic sampling and OLB. The location for bronchoalveolar lavage (BAL) sampling was selected on the basis of HRCT findings, or on a chest X-ray when HRCT was unavailable. BAL was performed by introducing 200 ml of sterile warm (37°C) saline solution into a lung subsegment and aspirating it back in four 50-ml aliquots. The first aliquot returned (bronchial fraction) was discarded. Each specimen was sent for bacterial examination for *Legionella*, *Mycoplasma pneumoniae*, *Pneumocystis carinii*, and *Mycobacteria*, and for fungal and virological (cytomegalovirus, influenza virus, parainfluenza virus, adenovirus, herpes simplex virus, respiratory syncytial virus, and coxsackie virus) analyses. Specimens were also sent for cytology and iron stain analysis. BAL results were deemed positive when at minimum one microorganism grew to a concentration of more than 10^4 ^colony-forming units/ml. All procedures were performed within 24 hours of OLB.

### Open lung biopsy

OLB was performed in an operating room or at the bedside in an ICU by an experienced thoracic surgeon. Bedside OLB was indicated when the FiO_2 _used reached 1 with an applied positive end-expiratory pressure (PEEP) of at least 12 cmH_2_O. With regard to mechanical ventilator settings to prevent air leakage, PEEP was immediately reduced 2 cmH_2_O from the baseline level after surgery. Pulmonary tissue was harvested from a site considered new or from a progressive lesion identified by chest HRCT or chest X-ray.

Each tissue specimen was cultured and examined by a pulmonary pathologist.

### Data collection

Medical records from these 41 patients were reviewed and analyzed for the following data: age; gender; Acute Physiology and Chronic Health Evaluation (APACHE) II scores at admission to the ICU; acute lung injury (ALI) scores, PEEP, and PaO_2_/FiO_2 _ratio at ARDS diagnosis; dates of ARDS onset, respiratory failure, intubation, and biopsy; underlying diseases; diagnostic tests before biopsy; and medications at time of biopsy. Results regarding complications of biopsy, pathological diagnosis, and postoperative therapeutic changes (addition or removal of drugs) were also analyzed. Outcome parameters, including ICU and hospital survival rates and cause of death, were also evaluated.

### Statistical analysis

For normally distributed data, values are reported as means ± SD. Student's *t *tests were used to compare normally distributed continuous variables. Differences between subgroups were compared by using the χ^2 ^test or Fisher's exact test when the expected number of events was less than five. The significance level (α) for all statistical tests was set at 0.05, and *p *< 0.05 was considered statistically significant.

## Results

Sixty-eight patients underwent OLB for ARDS evaluation during the study period, of whom 27 were excluded because the duration between intubation and OLB exceeded seven days. A total of 41 patients were enrolled. Table 1 lists the baseline characteristics of the patients studied. Twenty-four patients (59%) were immunocompetent and 17 patients (41%) were immunocompromised. Causes of immunocompromise status were hematological malignancy in 10 patients and solid tumors in four patients (three had bronchogenic cancers and one had breast cancer), HIV infection in two patients and renal transplantation in one. The duration from intubation to OLB for these 41 patients was 3.0 ± 1.9 days (mean ± SD; range 1 to 7).

BAL was performed 24 hours before OLB. Findings of BAL were compatible with pathological diagnosis for only four patients with diagnoses of bacterial pneumonia, mycobacterial tuberculosis, cytomegalovirus pneumonitis, and *Pneumocystis carinii *pneumonia. Twenty-two patients (54%) had chest HRCT before OLB to identify an appropriate biopsy site. For the remaining 19 patients who did not undergo chest HRCT, OLBs were performed from the right middle lobe in 12 patients and from the lingular lobe in seven patients.

Of the 41 patients, 26 (63%) underwent OLB in an operating room and 15 (37%) received bedside OLB in an ICU. Video-assisted thoracotomy was performed in eight patients, and the remaining patients underwent limited anterior thoracotomy. No intra-operative complication occurred, and eight patients (20%) had postoperative complications (less than seven days after the operation). Two patients developed transient hypotension after OLB and regained normal status after fluid resuscitation and vasopressor treatment for 12 hours. Two patients had pneumothorax diagnosed by chest X-ray and required a chest tube with low-pressure suction (10 cmH_2_O) drainage for 24 hours after OLB. Two patients had subcutaneous emphysema localized in the chest area after OLB, which resolved spontaneously in two days. Additionally, two patients had bronchopleural fistula with persistent air leaking from the operative chest tube for at least one day and did not need further surgery. Although six of these eight patients (two with transient hypotension, one with pneumothorax, one with subcutaneous emphysema and two with bronchopleural fistula) died, no surgical complication resulted directly in death. The incidence of postoperative complication was 15% (4/26) and 27% (4/15) for patients undergoing OLB in an operating room or at the bedside in an ICU, respectively. Complication rates were not significantly different between these two groups (*p *= 0.3799).

All biopsies provided sufficient data for pathological diagnosis (diagnostic yield 100%). The specimens obtained during OLB were sent for tissue culturing (for both bacteria and viruses); all culture results were negative. Pathological diagnoses were subdivided into specific and nonspecific categories. Eighteen patients (44%) had specific diagnoses established by OLB, and 23 (56%) had nonspecific diagnoses (Table 2).

Overall, OLB findings led to alteration therapy for 30 of 41 patients (73%). After OLB, 18 patients were administrated high-dose corticosteroid therapy (1 g/day methylprednisolone in divided doses for three days) and seven patients were treated with low-dose corticosteroid therapy (2–3 mg/kg per day methylprednisolone in divided doses). Three patients received co-trimoxazole for *Pneumocystis carinii *pneumonia. Antibiotics were changed in one patient and discontinued in one patient on the basis of pathological findings. Treatment was not changed in 11 of 41 patients (27%).

Table 3 presents comparative results for patient characteristics, complication rates, alterations in treatment, and survival rates of patients with specific and nonspecific pathological diagnoses by OLB. The rate of treatment alteration was higher in the nonspecific pathological diagnosis group than in that with a specific diagnosis (56% versus 87%; *p *= 0.0243). No other significant differences between these two groups were noted.

Twenty-one patients died in the ICU, resulting in an ICU survival rate of 49% (20/41). The hospital survival rate was the same as the ICU survival rate. Multiple organ dysfunction syndrome was the leading cause of death in 10 patients, followed by septic shock in nine patients, hypovolemic shock in one patient and acute myocardial infarction in one patient. Table 4 presents comparative results of patient characteristics and outcomes for survivors and nonsurvivors. No significant differences were observed between survivors and nonsurvivors for baseline data, such as age, gender, severity of illness, complication rate, and treatment alteration rate, between these two groups. Significantly more immunocompromised patients were in the survivor group than in the nonsurvivor group (60% vs 24%; *p *= 0.0187).

Comparisons between immunocompromised and immunocompetent patients (Table 5) showed that immunocompromised patients were younger (*p *= 0.0004) and had lower ALI scores (*p *= 0.0045). Furthermore, immunocompromised patients had better hospital survival rates than immunocompetent patients (71% versus 33%; *p *= 0.0187).

## Discussion

This study showed that OLB is an acceptably safe and useful procedure for some selected patients with early-stage ARDS. The treatment alteration rate was higher in patients with ARDS with nonspecific pathological diagnoses than in those with specific diagnoses.

In recent studies of patients with ARDS [7,8], OLB was employed relatively late, and the time from intubation to OLB was considerable (5 to 89 days in the study by Papazian and colleagues, and 0 to 25 days in the study by Patel). In the present study, OLB was performed within one week of intubation (3.0 ± 1.9 days), substantially earlier than in the previous two studies.

Patel and colleagues [8] reported that the BAL results predicted OLB findings in only two of 57 patients. The indication for OLB in the present study was suspected non-infectious ARDS, with no obvious etiology on the basis of clinical presentations. Of the 41 patients in our study, BAL results were compatible with the pathological diagnosis in only four patients. Most patients obtained a new diagnosis based on the OLB results, resulting in altered treatment. These findings suggest that the clinical characteristics used to indicate the application of OLB in addition to BAL was appropriate.

Numerous pulmonary disease entities can result in ARDS; however, the typical pulmonary pathology of ARDS is diffuse alveolar damage (DAD) in either acute or fibroproliferative stages. For patients with ARDS undergoing OLB, Patel and colleagues [8] identified the diagnostic rates of DAD and non-DAD as 40% (23/57) and 60% (34/57), respectively. The non-DAD diagnosis rate was higher in this study than that obtained by Patel and colleagues (71% versus 60%). Early OLB can obtain unexpected pathological diagnoses other than DAD and can facilitate effective treatment for patients with early-stage ARDS.

Specific diagnosis rates based on OLB findings vary among studies of patients with different disease entities. The specific diagnostic rates in a review by Cheson and colleagues were 21 to 68% in immunocompetent patients and 37 to 95% in immunocompromised patients [11-14]. In this study, specific and nonspecific diagnostic rates were 44% (18/41) and 56% (23/41), respectively, and specific diagnostic rates for immunocompetent and immunocompromised patients were 33% (8/24) and 59% (10/17), respectively. Although not statistically significant (*p *= 0.1052), the specific diagnostic rate between immunocompetent and immunocompromised patients was similar to that in previous studies, indicating that OLB obtains a high percentage of specific pathological diagnoses for immunocompromised patients.

In this study, the rate of therapy alterations after OLB was 73% (30/41) and was not lower than those in previous reports (range 59 to 75%) [10,14,15]. For groups with nonspecific and specific pathological diagnoses, the rate of changed therapy was higher in the nonspecific group (87% versus 56%; *p *= 0.0243). This analytical finding resulted from a large number of patients with nonspecific pathological diagnoses undergoing corticosteroid treatment as a rescue or anti-inflammatory therapy after excluding potential active infection, such as the fibroproliferative stage of DAD [16-18], interstitial pneumonitis, nonspecific interstitial pneumonitis, and organizing pneumonia. Early OLB can achieve diagnoses other than fibrosis that are potentially treatable with corticosteroid. Furthermore, the recent study by the ARDS Clinical Trials Network [19] did not support the routine use of methylprednisolone in patients with persistent ARDS (at least seven days after the onset) and suggested that methylprednisolone therapy might be harmful when initiated more than two weeks after the onset of ARDS. The duration of ARDS before corticosteroid treatment interacted significantly with survival.

For immunocompromised patients, some studies [11,20] suggested that OLB is advantageous for diagnosis and for treatment alteration but that its benefit to survival remains unclear. McKenna and colleagues [21] found that for immunocompromised patients, early OLB (average 3.6 days after admission) benefited the histological diagnosis of interstitial pneumonitis treated with steroids; however, OLB did not improve clinical outcome for all patients. The overall mortality rate was 51% (21/41) in the present study, which is similar to that obtained in previous reports (range 47 to 50%) [7,8]. More immunocompromised patients were in the survivors group and had a better survival rate than the immunocompetent patients (60% versus 24%; *p *= 0.0187); the young age and low ALI scores of immunocompromised patients probably accounted in part for their better outcome. Furthermore, the enhanced survival rate of immunocompromised patients might be attributed to more immunocompromised patients (9/13; 69%) than immunocompetent patients (9/17; 53%) receiving high-dose corticosteroid therapy after active infection had been excluded by OLB. Various pulmonary conditions such as infection, disease progression, therapeutic reaction, new and unrelated pathologies, or a combination of these can be present in immunocompromised patients [21,22]. For diagnostic yield and adequate treatment, early OLB has been considered to be a reliable diagnostic modality, providing an early and accurate etiological diagnosis in immunocompromised patients.

Operative complication rates reported for OLB in patients with ARDS have ranged from 17 to 39% [7,8,10]. In this study, the overall rate of OLB postoperative complications was 20% (8/41). In the late fibrotic stage, lung parenchyma is stiffer than in the earlier exudative or fibroproliferative stages of ARDS. Although operative complications are multifactorial, early OLB in non-stiff lungs (less fibrosis in the present study than in other reports) may account for the low surgical complication rate in this study. Of the 41 patients in the present study, 15 could not be transported to an operating room because they were being administered 100% O_2 _and a high PEEP; consequently, OLB was performed at the bedside in the ICU. No intra-operative complications or exacerbation of oxygenation and hemodynamics occurred, even in patients with ARDS with severe hypoxemia. Of these 15 patients, four developed postoperative complications of hypotension, pneumothorax, subcutaneous emphysema, and bronchopleural fistula, respectively. No death was attributable to OLB. The risk for complications due to OLB in early-stage ARDS was therefore acceptable, even for the most critically ill patients with severe hypoxemia.

Several limitations of this study should be considered. First, because of its retrospective nature our study cannot directly address the question of whether early OLB has a survival benefit. However, understanding of a specific etiology would permit the initiation of specific therapy assuming that such a therapy is available. Many of the diagnoses found in this study (such as metastatic malignancy, infectious pneumonia and hypersensitivity pneumonitis) may have an established positive therapeutic effect on outcome. Second, the result of this study cannot be generally applied to all patients with ARDS. The decision to perform OLB was not made at random and the patients referred for OLB were unlikely to be a representative sample of our ARDS population. This selection bias of patients and intensivists would be expected to increase the possibility of an alternative intervention. A third limitation is that some specific diagnosis such as viral pneumonitis may be underdiagnosed because its identification depends on the availability of laboratory facilities. A standardized comprehensive microbiological examination of BAL before OLB should be established.

## Conclusion

This retrospective study demonstrates that OLB had a high diagnostic yield rate and an acceptable complication rate for some selected patients with early-stage ARDS. The rate of treatment alteration was higher in patients with nonspecific pathological diagnoses than in those with specific pathologically diagnosed ARDS. Further prospective, randomized and control studies should investigate the appropriate indication and effect of OLB on outcome in patients with ARDS.

## Key messages

• 	Open lung biopsy is an acceptably safe diagnostic procedure for some selected early-stage patients with acute respiratory distress syndrome.

• 	In patients with early-stage acute respiratory distress syndrome of suspected non-infectious origin, open lung biopsy may have a high diagnostic yield rate.

• 	The role of open lung biopsy in patients with acute respiratory distress syndrome needs to be investigated in prospective, randomized and controlled clinical trials.

## Abbreviations

ALI = acute lung injury; ARDS = acute respiratory distress syndrome; BAL = bronchoalveolar lavage; DAD = diffuse alveolar damage; FiO_2 _= fraction of inspired oxygen; HRCT = high-resolution computed tomography; ICU = intensive care unit; OLB = open lung biopsy; PaO_2 _= arterial partial pressure of oxygen; PEEP = positive end-expiratory pressure.

## Competing interests

The authors declare that they have no competing interests.

## Authors' contributions

KCK, YHT, YKW, NHC, and MJH collected and analyzed the data. SFH reviewed the pathological specimens. CCH conceived and coordinated the study. All the authors contributed to, read and approved the final manuscript.

## Figures and Tables

**Table 1:** Baseline characteristics

Characteristic	Value
Age (years)	55 ± 17
Male sex	28 (68%)
APACHE II score	22 ± 4.9
ALI score	3.1 ± 0.4
PEEP, cmH_2_O	11.1 ± 3.1
PaO_2_/FiO_2_, mmHg	116 ± 43
BAL before biopsy	32 (78%)
HRCT before biopsy	22 (54%)
Underlying disease	
Immunocompetent	24 (59%)
Immunocompromised	17 (41%)
Hematological malignancy	10
Solid cancer	4
HIV infection	2
Renal transplantation	1
Days from intubation to biopsy	3.0 ± 1.9

APACHE, Acute Physiology and Chronic Health Evaluation; ALI, acute lung injury; PEEP = positive end-expiratory pressure; PaO_2_, arterial partial pressure of oxygen; FiO_2_, fraction of inspired oxygen; BAL, bronchoalveolar lavage; HRCT, high-resolution computed tomography. Data are presented as mean ± SD or *n *(%).

**Table 2:** Pathological diagnoses

Diagnosis	*n*
Specific	18 (44%)
*Pneumocystis carinii *pneumonia	4
Metastatic malignancy	3
Bacterial pneumonia	3
Fungal infection	2
Unusual interstitial pneumonitis	2
Hypersensitive pneumonitis	1
Mycobacterial tuberculosis	1
Cytomegalovirus pneumonitis	1
Metastatic calcification	1
Nonspecific	23 (56%)
Diffuse alveolar damage	12
Acute stage	2
Fibroproliferative stage	10
Fibrosis	4
Interstitial pneumonitis	3
Pulmonary edema	2
Nonspecific interstitial pneumonitis	1
Organizing pneumonia	1

**Table 3:** Patient characteristics for specific and nonspecific diagnoses

Characteristic	Specific (*n *= 18)	Nonspecific (*n *= 23)	*p*
Age	50 ± 20	58 ± 10	0.0943
Sex			
Male	14 (78%)	14 (61%)	0.2482
Female	4 (21%)	9 (39%)	
APACHE II score	21.8 ± 5.0	21.7 ± 4.9	0.95
ALI score	3.1± 0.3	3.2 ± 0.4	0.3
PaO_2_/FiO_2_, mmHg	120.0 ± 42.2	113.6 ± 44.8	0.6
Immune status			
Compromised	10 (56%)	7 (30%)	0.1052
Competent	8 (42%)	16 (70%)	
Complication rate	4 (22%)	4 (17%)	0.6985
Treatment alteration rate	10 (56%)	20 (87%)	*0.0243*
Hospital survival rate	6 (33%)	14 (61%)	0.08

APACHE, Acute Physiology and Chronic Health Evaluation; ALI, acute lung injury; PaO_2_, arterial partial pressure of oxygen; FiO_2_, fraction of inspired oxygen. Data are presented as mean ± SD or *n *(%); *p *values in italics are statistically significant.

**Table 4:** Patient characteristics for survivals and nonsurvivals

Characteristics	Survivors (*n *= 20)	Nonsurvivors (*n *= 21)	*p*
Age	51 ± 15	59 ± 18	0.1621
Sex			
Male	14 (70%)	15 (71%)	0.92
Female	6 (30%)	6 (29%)	
APACHE II score	20.6 ± 3.7	23.0 ± 5.7	0.12
ALI score	3.0 ± 0.4	3.2 ± 0.3	0.1
PaO_2_/FiO_2_, mmHg	126.2 ± 44.2	107.0 ± 41.2	0.1571
Immune status			
Compromised	12 (60%)	5 (24%)	*0.0187*
Competent	8 (40%)	16 (76%)	
Complication rate	2 (10%)	6 (29%)	0.1336
Diagnosis			
Specific	6 (30%)	12 (57%)	0.08
Nonspecific	14 (70%)	9 (43%)	
Treatment alteration rate	16 (80%)	14 (68%)	0.3355

APACHE, Acute Physiology and Chronic Health Evaluation; ALI, acute lung injury; PaO_2_, arterial partial pressure of oxygen; FiO_2_, fraction of inspired oxygen. Data are presented as mean ± SD or *n *(%); *p *values in italics are statistically significant.

**Table 5:** Patient characteristics for different immune status

Characteristics	Competent (*n *= 24)	Compromised (*n *= 17)	*p*
Age	62 ± 15	45 ± 13	*0.0004*
Gender			
Male	17 (71%)	12 (71%)	0.9864
Female	7 (29%)	5 (29%)	
APACHE II score	22.1 ± 5.1	21.4 ± 4.9	0.6
ALI score	3.3 ± 0.4	2.9 ± 0.3	*0.0045*
PaO_2_/FiO_2_, mmHg	108.8 ± 44.1	130.2 ± 40.1	0.1855
Complication rate	5 (21%)	3 (18%)	0.7998
Diagnosis			
Specific	9 (38%)	9 (53%)	0.3263
Nonspecific	8 (47%)		
Treatment alteration rate	17 (71%)	13 (77%)	0.6882
Hospital survival rate	8 (33%)	12 (71%)	*0.0187*

APACHE, Acute Physiology and Chronic Health Evaluation; ALI, acute lung injury; PaO_2_, arterial partial pressure of oxygen; FiO_2_, fraction of inspired oxygen. Data are presented as mean ± SD or *n *(%); *p *values in italics are statistically significant.
